# Spatial and Temporal Variation of Precipitation Drives the Genome Size Variation in *Scolopendra* in Chinese Mainland

**DOI:** 10.1002/ece3.70580

**Published:** 2024-11-18

**Authors:** Kai Zhang, Buddhi Dayananda, Yifei Liu, Zhigang Hu, Lin Zhang

**Affiliations:** ^1^ Hubei Shizhen Laboratory, College of Pharmacy Hubei University of Chinese Medicine Wuhan China; ^2^ School of Agriculture and Food Sciences The University of Queensland Brisbane Queensland Australia; ^3^ Hubei Shizhen Laboratory, Hubei Key Laboratory of Theory and Application Research of Liver and Kidney in Traditional Chinese Medicine, School of Basic Medical Sciences Hubei University of Chinese Medicine Wuhan China

**Keywords:** ecological factor, genome size, precipitation niche breadth, *Scolopendra*

## Abstract

Genome size is an adaptive trait, and its variations influence the organismal phenotype and fitness. In this study, we propose a hypothesis linking variations in genome size within *Scolopendra* to ecological factors. To test this hypothesis, we employed flow cytometry to estimate genome size in seven *Scolopendra* species from Chinese mainland. Subsequently, we reconstructed the phylogenetic relationship of these species using the cytochrome *c* oxidase subunit 1 gene and conducted phylogenetic comparative analysis to assess the relationships between genome size and niche breadth or 19 bioclimatic variables. Our findings indicate the following: (1) genome size in *Scolopendra* can be categorized into three groups, similar to the phylogenetic clades; (2) there is a negative correlation between genome size in *Scolopendra* species and the precipitation niche breadth of species; (3) the estimated divergence time of *Scolopendra* dates back 153 Mya, during the Jurassic period. We assume that consistent aridity geological periods may promote the evolution of *Scolopendra* species with a large genome size, whereas rapidly fluctuating humidity geological periods may have the opposite effect.

## Graphical abstract

Genome size in *Scolopendra* can be categorized into three groups that align with the phylogenetic clades; there is a negative correlation between genome size in *Scolopendra*s and the precipitation niche breadth of species; the estimated divergence‐time of *Scolopendra* dates back 153 Mya, during the Jurassic period.

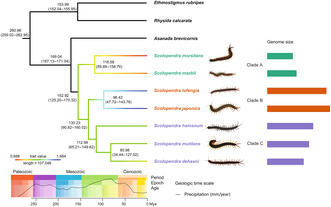

## 1 Introduction

Genome size demonstrates sibnificant diversity across various animal species, ranging from 0.01956 (*Pratylenchus coffeae*) to 129.09774 GB ( *Protopterus aethiopicus* ) with nearly a 6600‐fold changes of variation (Gregory 2005a). Several hypotheses have been put forth to explain the factors driving variations in genome size (Gregory 2005b; Lynch and Walsh 2007); this diversity arises from various genetic mechanisms, including insertions, deletions, and duplications (Sun, Lopez Arriaza, and Mueller 2012; Shao, Han, and Peng 2019). Furthermore, the variations in genome size can primarily be attributed to two important mechanisms: whole‐genome duplication events and the proliferation of noncoding elements (Neiman et al. 2015; Dufresne and Jeffery 2011). A strong link exists between the organism's genome size and its phenotype, with several ecological and physiological factors exerting synergistic effects on this relationship (Gregory 2002; Iannucci et al. 2022). It has been proposed that genome size is associated with a range of physiological characteristics, which are affected by both intrinsic factors, like nucleus size (Saha et al. 2023), cell size (Hessen, Daufresne, and Leinaas 2013; Malerba, Ghedini, and Marshall 2020), cell division rate (Gregory 2002; François, Samuel, and Vahid 2018), and metabolic rate (Gardner, Laurin, and Organ 2020), as well as extrinsic factors like ecological constraints (Guignard et al. 2016; Yu et al. 2020; Chen et al. 2021; Wang et al. 2024).

Furthermore, genome size is associated with the ecological distribution of species. A larger genome size is associated with species inhabiting slower‐developing habitats with permanent water sources (Sclavi and Herrick 2019; Lertzman‐Lepofsky, Mooers, and Greenberg 2019). Organisms with a wide ecological tolerance tend to have larger genome sizes (Bennett 1987; Beaton and Hebert 1989), and freshwater and eurybiotic species generally have larger genomes compared to marine and stenobiotic species (Ebeling, Atkin, and Setzer 1971; Hardie and Hebert 2003). Complex associations exist between genome size and ecological factors in various groups of organisms, for instance, the Caesalpinia group tends to have larger genome sizes at higher latitudes (Souza et al. 2019), Actinopterygii group exhibits a larger genome size in regions with higher temperature (Smith and Gregory 2009), amphibians, arthropods, fish, and reptiles often have a larger genome size but maintain a high lower critical thermal limit (Leiva, Calosi, and Verberk 2019), as poikilotherms generally possess larger genomes in colder regions (Xia 1995) and a negative relationship between niche rate and genome size in Caudata (Sclavi and Herrick 2019). In frogs, genome size is indirectly influenced by humidity (Liedtke et al. 2018). The *Streptanthus* species tend to possess a smaller genome size in regions characterized by significant climate seasonality (Cacho et al. 2021), and Liliaceae and *Eragrostis* have a smaller genome size in high precipitation regions (Carta and Peruzzi 2016; Hutang et al. 2023). Climatic niche constraints may have played a key role in the evolution of genome size (Saha et al. 2023).

The Arthropoda phylum stands out as the most diverse group of animals and exhibits notable patterns of gene loss (Fernández and Gabaldón 2020; Guijarro‐Clarke, Holland, and Paps 2020). In Crustacea, there is a positive relationship between the maximum observed latitude and depth and the size of the genome (Alfsnes, Leinaas, and Hessen 2017). In Insecta, the genome size mirror various life cycle strategies, including life history, mode of development, and habitat preferences (Alfsnes, Leinaas, and Hessen 2017). In Myriapoda, the influence of transposable elements looms large in the evolution of genome size (So et al. 2022). *Scolopendra*, with more 100 species, is one of the oldest extant terrestrial arthropods, ranging from all continents except Antarctica, and plays a crucial role in ecosystems, including pest control, soil health improvement, nutrient cycling, and support for biodiversity (Wiegand 2024). According to the Global Biodiversity Information Facility (GBIF, accessed July 20, 2023), there are 15 species of *Scolopendra* recorded in China, whereas previous studies have only examined 14 of these species (Kang et al. 2017).

Compared to the genome size within the genus *Scolopendra*, integrating phylogenetic relationships can help unravel the mystery of genome size variation (Hjelmen and Johnston 2017). This, in turn, can shed light on the influence of ecological factors on species evolution (Liedtke et al. 2018) and aid in understanding the evolutionary drivers operating across different timescales (Gregory 2005b). To tackle these inquiries, this study investigated the evolutionary history of *Scolopendra* by constructing a phylogenetic framework. Simultaneously, we explore the correlation between *Scolopendra* genome size and ecological factors. To explore these connections, we suggest the hypothesis that genome size varies with climate factors. To test this hypothesis, we employ flow cytometry to estimate genome sizes and reconstruct the phylogenetic relationship among seven *Scolopendra* species. Subsequently, we conducted a study to examine the relationship between genome size and climate factors employing a phylogenetic generalized least squares (PGLS) model.

## 2 Materials and Methods

### 2.1 Sample Collection and *COX1* Sequencing

We collected seven *Scolopendra* species, including *Scolopendra dehaani* , *S. hainanum*, *S. japonica* , *S. lufengia* , *S. mazbii* , *S. mutilans*, and *S. morsitans* from Chinese mainland, extracted the DNAs from the leg muscle of these seven species and amplified the mitochondrial cytochrome c oxidase subunit 1 (*COX1*, 689 bp) using the primer LCO1490/HCO2198 (Folmer et al. 1994), and then sequenced to confirm the species (Figure S1).

### 2.2 Phylogenetic Analyses

The maximum clades credibility tree from divergence time rooted phylogenetic analyses was estimated by BEAST v.2.0 (Bouckaert et al. 2014) based on the *COX1* gene. We selected three times to estimate the divergence time of *Scolopendra* species: (1) 261 Mya, the divergence time among *Rhysida*, *Ethmostigmus*, *Asanada*, and *Scolopendra*, (2) 169 Mya as the divergence time between *Asanada* and *Scolopendra*, and (3) 154 Mya, as the divergence time between *Rhysida* and *Ethmostigmus*. The analysis incorporated the use of the relaxed lognormal clock model. For the tree prior, the standard Yule speciation process was specifically chosen. In this analysis, the clock models and topologies of individual data partitions were linked, while the substitution parameters remained unlinked across the partitions. For the BEAST analyses, we used four replicate searches with 100 million generations each, retaining trees every 10,000 generations. We compared results of independent runs using Tracer v.1.7.2 (Rambaut et al. 2018) to ensure that the chains were converging and mixing adequately. Subsequently, we excluded the initial 10% of sampled generations from each run as burn‐in. From the remaining results, we employed the outcomes from the runs (likelihood = −4165.797, ESS = 2505). Then we obtained the precipitation data for the past 540 million years from Li's work (Li et al. 2022).

### 2.3 Genome Size Estimation

We interpreted 2C DNA content as genome size in this study. We measured the genome size of all samples employing two reference standards: *Mus musculus* (2.728 GB, NCBI reference genome) and *Channa argus* (0.613 GB) (Xu et al. 2017). Samples were considered to have a reliable estimate when the coefficient of variation (CV) was less than 5% (Table 1, Figures S2 and S3). Our samples underwent a meticulous pretreatment procedure following Johnston's method (Johnston, Bernardini, and Hjelmen 2019), and all samples were freshly prepared on the day of execution. Initially, each sample was individually assessed using a flow cytometer to observe the relative fluorescence intensity of their genomic DNA. This initial assessment allowed us to determine the approximate fluorescence peak positions for all samples. Subsequently, the samples to be tested were mixed with reference samples, and 10,000 particles were collected for each group of samples. The fluorescence in the FL2 channel, along with the fluorescence intensity of PI, was recorded, and the preliminary results were stored using Cflow Plus software. Our flow cytometry analysis of *C. argus* samples revealed distinct and well‐defined peaks that closely matched the peak positions of the test samples. Based on this observation, we chose to employ the *C. argus* as a reference sample to estimate the genome size of the respective test samples. During the analysis of peak positions using flow cytometry, it was noted that the peak positions of *S. mazbii* and *S. morsitans* overlapped with those of *C. argus* . Consequently, we proceeded to estimate the genome size of these two *Scolopendras* using *M. musculus* as a reference and subsequently adjusted the final results based on *C. argus* data. Following the processing of the converted FCS files using specialized software, we further analyzed these files using flowjo 10.8.1 software. The fluorescence intensity of G1 phase was collected using the FL2 channel, allowing for genome size prediction by leveraging the qualitative and quantitative properties of fluorescent dyes in conjunction with the structure of the DNA double‐stranded carbon framework. In essence, the strength of the fluorescent signal exhibited a positive correlation with the amount of DNA bound, with increased DNA content resulting in stronger fluorescence intensity (Dolezel et al. 2003). Ultimately, we calculated the genome size of the samples using the following formula:

GSs=DNAs/DNAr×GSr

GSs, genome size of samples; DNAs, DNA fluorescence intensity of samples; DNAr, DNA fluorescence intensity of reference samples; GSr, genome size of the reference sample.

**TABLE 1:** Flow cytometry predicts genome size in seven *Scolopendras*.

Species	DNA fluorescence intensity of samples	Coefficient of Variation of samples	Estimated genome size of samples (GB)
*Scolopendra dehaani*	537,878.76	3.76%	0.98
*Scolopendra hainanaum*	672,931.19	2.82%	1.23
*Scolopendra japonica*	930,219.91	3.01%	1.68
*Scolopendra lufengia*	856,232.71	2.92%	1.59
*Scolopendra mazbii*	404,186.86	2.38%	0.79
*Scolopendra morsitans*	356,906.40	3.45%	0.69
*Scolopendra mutilans*	607,933.33	1.94%	1.12

### 2.4 Environmental Data

We obtained species occurrence data from GBIF (accessed July 20, 2023, Yesson et al. 2007). We also obtained 19 bioclimatic variables from the WorldClim 2.1 (https://worldclim.org/) based on the resolution of 30 s (Fick and Hijmans 2017). ENMTools (Warren, Glor, and Turelli 2010) was used to filter sample points (Table S1) for each species to ensure that there was only one sample point at each 30 s raster. We compiled a dataset consisting of a total of 1269 sample points encompassing several *S. mutilans*, *S. dehaani* , *S. hainanum*, *S. morsitans* , and *S. japonica* . However, it is worth noting that *S. hainanum* had fewer than 50 sample points available for analysis. To assess the temperature niche breadth (TNB), we calculated the difference between the minimum value of the coldest month's temperature and the maximum value of the warmest month's temperature across all sampled localities. For our first niche breadth index, we focused on precipitation niche breadth (PNB) and selected an indicator that reflects spatial variation. This was achieved by subtracting the minimum annual precipitation values from the maximum annual precipitation values across all localities. For our second niche breadth index, we concentrated on precipitation and considered a species' PNB (P‐SNB). This index was derived from the difference between the maximum precipitation value during the wettest quarter and the minimum precipitation value during the driest quarter, incorporating both spatial and seasonal variations in precipitation (Table S2).

### 2.5 Data Analyses

We estimated the genome size phylogenetic signal with Blomberg's *K* in the *picante* package (Steven et al. 2010) and performed an ancestral state reconstruction of genome size in the *Phytools* package (Revell 2012) implemented in R 4.3.1. The value of Blomberg's *K* close to 0 indicates a weak or nonexistent phylogenetic structure, while values close to 1 are expected if the data follow a Brownian Motion (BM) evolution model (Blomberg, Garland, and Ives 2003). We performed both ordinary least squares (OLS) and PGLS with the *rms* package (Harrell Jr 2023) and *caper* package (Orme et al. 2023) in R 4.3.1, respectively. To assess the degree of the phylogenetic signal in our data, we employed Pagel's lambda (*λ*) as a measure. A *λ* value close to 0 indicates a lack of phylogenetic dependence or independence, while *λ* values near or equal to 1 indicate a strong phylogenetic signal in the dataset (Freckleton, Harvey, and Pagel 2002).

## 3 Results

**FIGURE 1:**
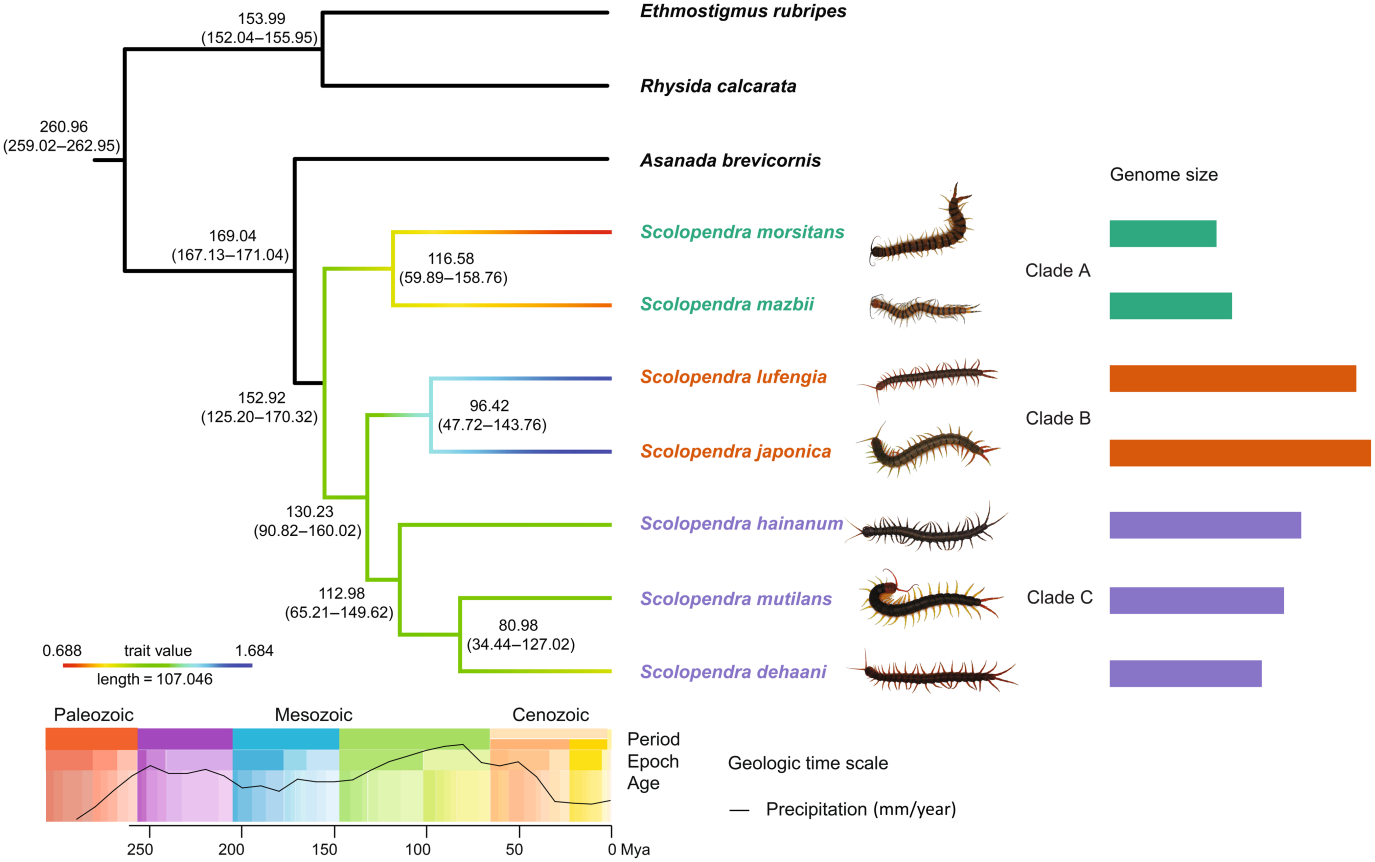
Results of the molecular clock and genome sizes of *Scolopendra*. The colors on different branches represent different genome sizes, and to make the results more intuitive, we used rectangles of different color lengths behind the species photos to show the differences in species and genome size on different branches. Green groups represent small genome size branches Clade A, orange branches represent large genome size branches Clade B, and purple groups represent medium genome size branches Clade C. The parentheses below each time point represent 95% credibility intervals from the considered analysis. We placed the geologic time scale and precipitation (mm/year) at the bottom of the graph.

Utilizing flow cytometry, we estimated the genome size of seven *Scolopendra* species in Chinese mainland, revealing an average genome size of 1.15 GB with a notable 2.43‐fold variation, ranging from 0.69 GB ( *S. morsitans* ) to 1.68 GB ( *S. japonica* ) (Figure 1, Figures S1 and S2 and Table 1), and intriguingly, the variation in genome size exhibited a discernible pattern among sister species, with the ancient genome size falling within the medium range.

The phylogenetic analysis unveiled a tree of *Scolopendra* species with three clades: (1) Clade A, including *S. mazbii* and *S. morsitans* , with a small genome size; (2) Clade C including *S. hainanum*, S. *mutilans*, and *S. dehaani* , with a medium genome size; and (3) Clade B, including *S. japonica* and *S. lufengia* , with a large genome size. The divergence time of *Scolopendra* was estimated at 153 Mya (Figure 1), with species in clade A diverging between clade B and clade C, during the Jurassic period (Figure 1).

**FIGURE 2:**
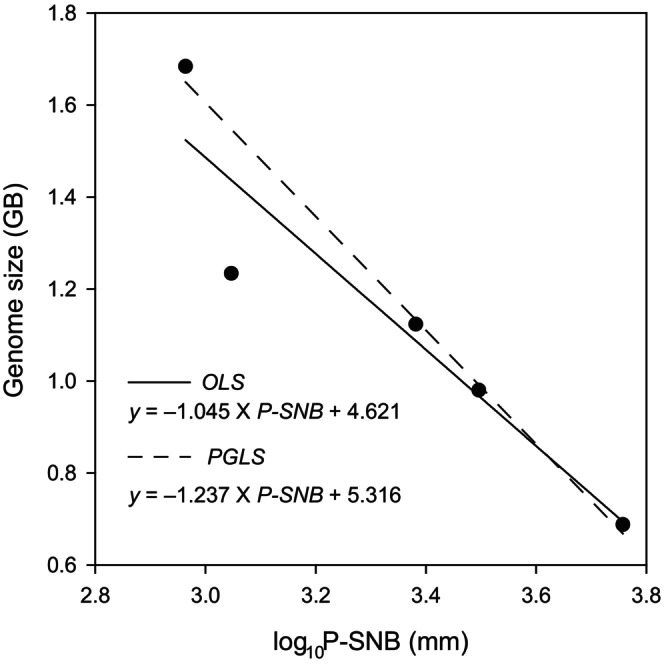
The relationship with phylogenetic signal between genome size and log_10_P‐SNB. The *x*‐axis represents Logarithm of species precipitation niche breadth and the *y*‐axis represents the genome size.

In our analysis, we did not observe a significant correlation between genome size and TNB, PNB, and 19 bioclimatic variables (all *p* > 0.05), except for P‐SNB (OLS: slope = −1.045, *r* ^2^ = 0.827, *F*_1,3_ = 20.48, *p* = 0.02, Figure 2, Table S4). Genome size (*K* = 0.955, *p* = 0.027, Table S3) presented a phylogenetic signal. There is a negative relationship between genome size and P‐SNB (PGLS: slope = −1.237, λ = 1.00, *r* ^2^ = 0.976, *F*_1,3_ = 146.3, *p* = 0.0012; Figure 2, Table S4).

## 4 Discussion

The study elucidates a negative correlation between genome size and P‐SNB among the seven *Scolopendra* species from Chinese mainland, whether OLS or PGLS analysis. The phylogenetic tree analysis revealed three clades among the *Scolopendra*, with species exhibiting similar genome size tendencies clustering into specific clades. The estimated divergence time, approximately 153 Mya, coincides with the Jurassic period marked by increasing precipitation.

Many species displayed robust phylogenetic signals, which could be linked to the heritable nature of genome size (de Ricqlès et al. 2008). When genome size did not evolve adaptively, the phylogenetic signal (*λ*) approached 1 (Pandit, White, and Pocock 2014). However, in *Eragrostis* species (Hutang et al. 2023), the phylogenetic signal significantly deviated from 1, indicating adaptive evolution (Gregory 2005b; Gregory and Johnston 2008; Losos 2008) and the adaptation of existing species to new niches while retaining some ancestral environmental features (Wiens and Graham 2005). In salamanders, a negative relationship with *λ* = 1 was observed between niche breadth and genome size (Sclavi and Herrick 2019), supporting the notion that, over extended evolutionary timeframes, changes in genome size in salamanders were coupled with adaptations to available habitats and niches for ancestral population dispersal. The PGLS analyses unveiled associations between habitat variations and genome size in Crustacea and Insecta species (Alfsnes, Leinaas, and Hessen 2017), further supporting the idea that genome size in arthropods may be influenced by phylogenetic relationships. We elucidated a “strong” phylogenetic signal in the genome size based on the incomplete taxon sampling; it suggests a potential association between genome size evolution and phylogenetic relationships. However, incomplete taxon sampling will be affecting the accuracy and robustness of phylogenetic signal analyses (Wiens and Tiu 2012).

Specific environmental conditions drive genome size evolution (Carta and Peruzzi 2016). Environmental factors (temperature or/and precipitation) shaped genome size variation as a traits of adaptation response (Sclavi and Herrick 2019; Liedtke et al. 2018; Leiva, Calosi, and Verberk 2019; Trávníček et al. 2019). Salamanders were found to have smaller genomes in rapidly fluctuating habitats (Lertzman‐Lepofsky, Mooers, and Greenberg 2019), while in extreme environment (dry or ephemeral), small genome sizes were associated with rapid developmental rates in frogs (Liedtke et al. 2018; Zeng, Gomez‐Mestre, and Wiens 2014). The P‐SNB reflected the spatial and temporal variation of precipitation, and there was a negative correlation between genome size and P‐SNB, whether OLS or PGLS analysis (Figure 2); it indicates that a species with a small genome size lives in the rapidly fluctuating habitats. Our result supported the hypothesis that species with smaller genomes size have a wider area (Pandit, White, and Pocock 2014) (Figure S4) and live in an unstable environment (Knight, Molinari, and Petrov 2005) (Figure 2). Although the relationship between genome size and precipitation is unclear in animals, coincidentally, our result was similar to it in plants. For instance, Liliaceae evolved larger genomes in humid climate (Carta and Peruzzi 2016), whereas *Streptanthus* displayed small genome size in regions with a greater climate seasonality (Cacho et al. 2021). In *Eragrostis*, larger genomes were associated with less precipitation (Hutang et al. 2023). While ploidy levels are known to strongly influence genome sizes in plants (Carta and Peruzzi 2016; Cacho et al. 2021; Hutang et al. 2023), their role in animals remains unclear. Notably, the study revealed that transposable elements (TEs) are a major contributor to genome size evolution in myriapods (So et al. 2022), with more than 420 Mb (47.01% of the genome) of TEs in *S. mutilans* (Zhang et al. 2024). Given the divergence time of 153 Mya and the negative correlation between genome size and P‐SNB, it is postulated that *Scolopendra* species could have evolved toward smaller genome size when transitioning from arid to humid conditions during the Jurassic period, coinciding with increased precipitation (Figure 1). Annual mean precipitation maintained a stable level about 1135 mm/year between 170 and 140 Mya; it increased from 1135 to 1284 mm/year between 130 and 80 Mya; then it decreased from 1284 to 1211 mm/year between 80 and 70 Mya (Li et al. 2022). The trend of annual mean precipitation from 170 to 70 Mya coupled the divergence time of the *Scolopendra* species. This work supported the large genome constraint hypothesis; the hypothesis postulated that species with a large genomes size are prevalent in the stable environment (Knight, Molinari, and Petrov 2005). For increase in the cell replication rate and a contraction of development periods, high temperature and aridity induced genome size toward to small genome size in anurans (Lertzman‐Lepofsky, Mooers, and Greenberg 2019; Liedtke et al. 2018). Regarding the genome size changes in other species, we speculate that the rapidly fluctuating habitats, coupled with an increase in annual mean precipitation, have led to a reduction in the genome size of *Scolopendra*.

## 5 Conclusions

In summary, our study has shed light on the intricate connections between genome size and climate factors in *Scolopendra*. We found that TNB, PNB, and 19 bioclimatic variables have no significant influence on the genome size variation. However, a notable pattern emerges, where species with smaller genome sizes tend to be associated with greater P‐SNB. Our future research would need more *Scolopendra* species to reveal the relationships between genome size evolution and climate niche.

## Author Contributions

**Kai Zhang:** data curation (equal), formal analysis (equal), resources (equal), visualization (equal), writing – original draft (equal), writing – review and editing (equal). **Buddhi Dayananda:** supervision (equal), writing – review and editing (equal). **Yifei Liu:** resources (equal), supervision (equal), writing – original draft (equal), writing – review and editing (equal). **Zhigang Hu:** funding acquisition (equal), project administration (equal), resources (equal), supervision (equal), writing – original draft (equal), writing – review and editing (equal). **Lin Zhang:** conceptualization (equal), data curation (equal), formal analysis (equal), funding acquisition (equal), methodology (equal), project administration (equal), resources (equal), software (equal), supervision (equal), visualization (equal), writing – original draft (equal), writing – review and editing (equal).

## Conflicts of Interest

The authors declare no conflicts of interest.

## Supporting information


**Figure S1.** Neighbor‐Joining tree were based on cytochrome c oxidase subunit 1 (*COX1*).
**Figure S2.** Flow cytometry results plotted on individual samples.
**Figure S3.** Flow cytometry results plotted on mixed samples.
**Figure S4.** Relationship between genome size and species distribution range.

**Table S1.** Sample latitude and longitude information and 19 bioclimatic variables for the five filtered *Scolopendra* species.
**Table S2.** TNB, PNB and P‐SNB for five *Scolopendra* species.
**Table S3.** Phylogenetic signal for genomics size calculated through Blomberg’s *K*.
**Table S4.**Statistics describing the relationships shown in Figure 2. Models were fitted using both OLS and PGLS regressions.

## Data Availability

Sequencing data were exported as individual FASTA files and have been deposited in Genbank (Metazoan Mitochondrial *COX1*) NCBI (https://www.ncbi.nlm.nih.gov/) under the accession code: SUB13720767 (OR413624–OR413630).
