# Supplementary material for: Spatial and Temporal Variation of Precipitation Drives the Genome Size Variation in Scolopendra in Chinese Mainland
Source: Ecol Evol. 2024 Nov 18;14(11):e70580. doi: 10.1002/ece3.70580 (PMC11573723; doi:10.1002/ece3.70580)
Supplement: Supplementary file 1 — Figure S1. Neighbor‐Joining tree were based on cytochrome c oxidase subunit 1 (COX1). Figure S2. Flow cytometry results plotted on individual samples. Figure S3. Flow cytometry results plotted on mixed samples. Figure S4. Relationship between genome size and species distribution range. [file ECE3-14-e70580-s001.docx]

Supplementary Materials for

**Precipitation niche breadth driven the genome size variation in *Scolopendra***

Kai Zhang^1^, Zezhi Shu^1^, Lingfeng Peng^1^, Mingyu Zhu^1^, Yifei Liu^1^, Buddhi Dayananda^2^, Zhigang Hu^1, *^, Lin Zhang ^3,4 *^

^1^ College of Pharmacy, Hubei University of Chinese Medicine, Wuhan 430065, China

^2^ School of Agriculture and Food Sciences, The University of Queensland, Brisbane, QLD 4072, Australia

^3^ School of Basic Medical Sciences, Hubei University of Chinese Medicine, Wuhan 430065, China

^4^ School of Ecology and Environmental Sciences, Yunnan University, Kunming 650500, China

*Correspondence: lzhangss@msn.com (L.Z.) and zghu0608@hbtcm.edu.cn (Z.H.)

Running title: the genome size variation in *Scolopendra*

Supplementary Figures
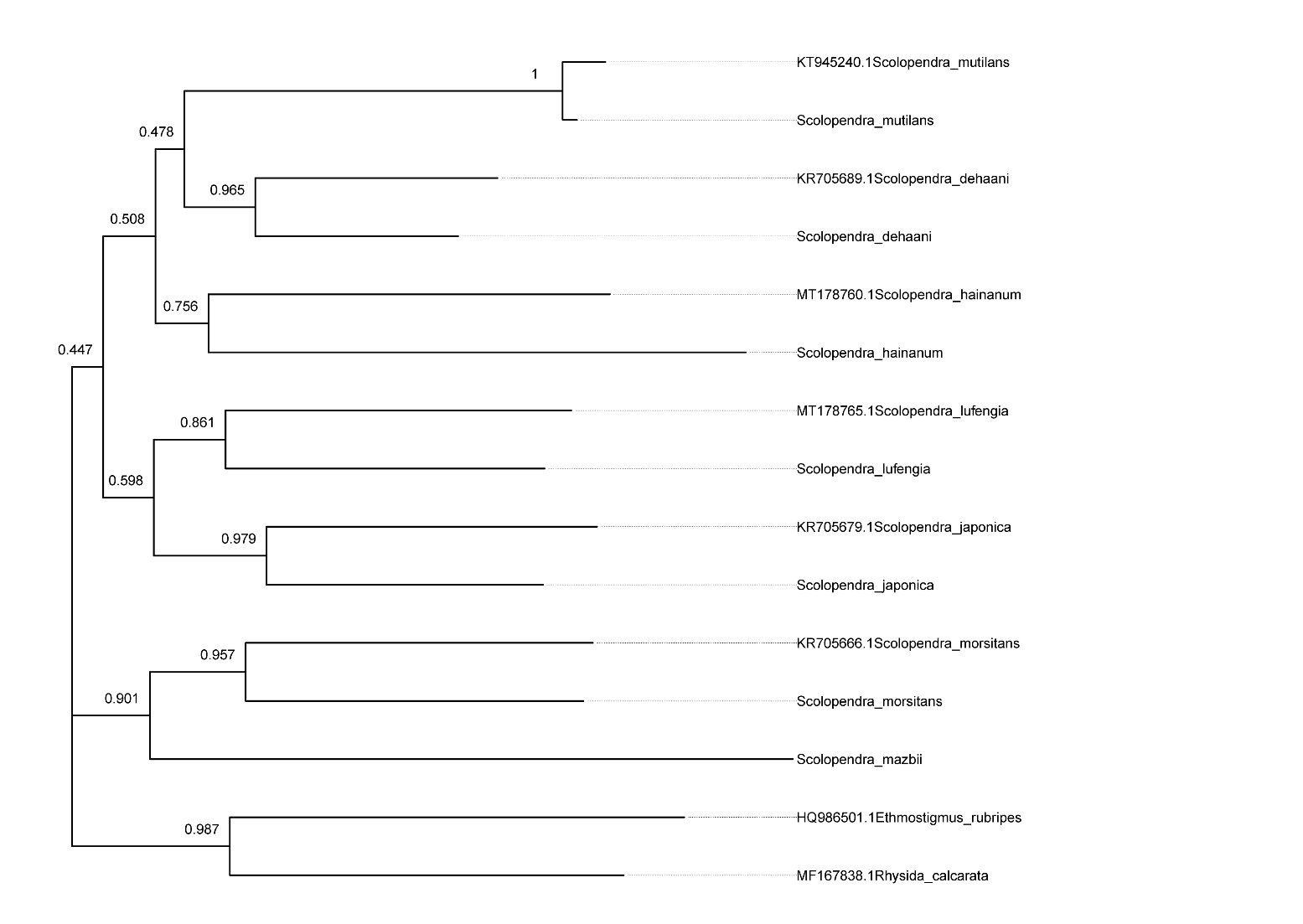


**Figure S1. Neighbor-Joining tree were based on cytochrome c oxidase subunit 1 (COX1).** We used Sequence ID+species name to name the data downloaded from NCBI, while the data only with species name is what we sequenced, The tree was rooted with *Ethmostigmus rubripes* and *Asanada brevicornis* (outgroup).


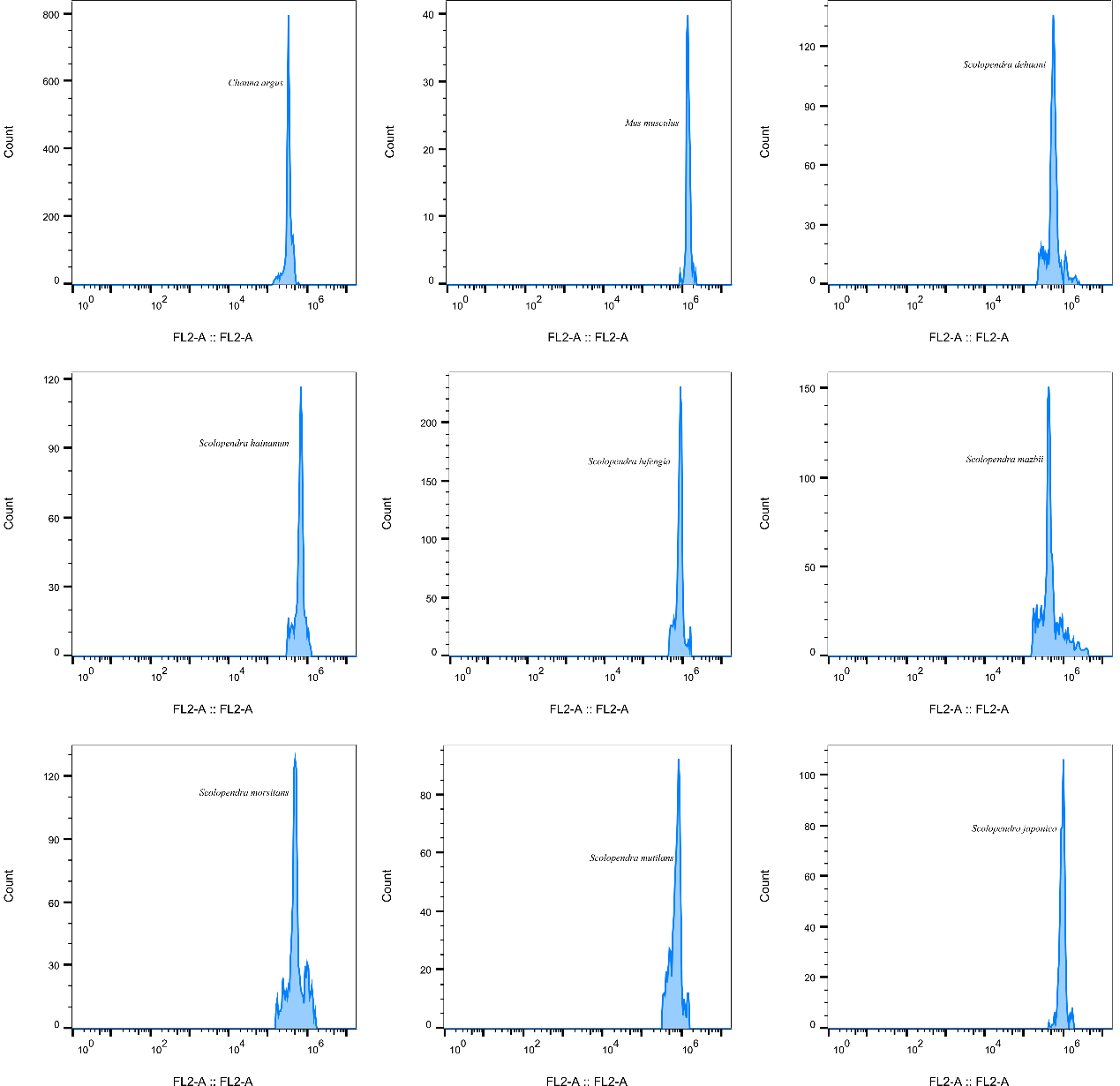


**Figure S2:** **Flow cytometry results plotted on individual samples.** Each peak represents the fluorescence intensity when injected individually with flow cytometry, and next to the peak we labeled the species name.

**
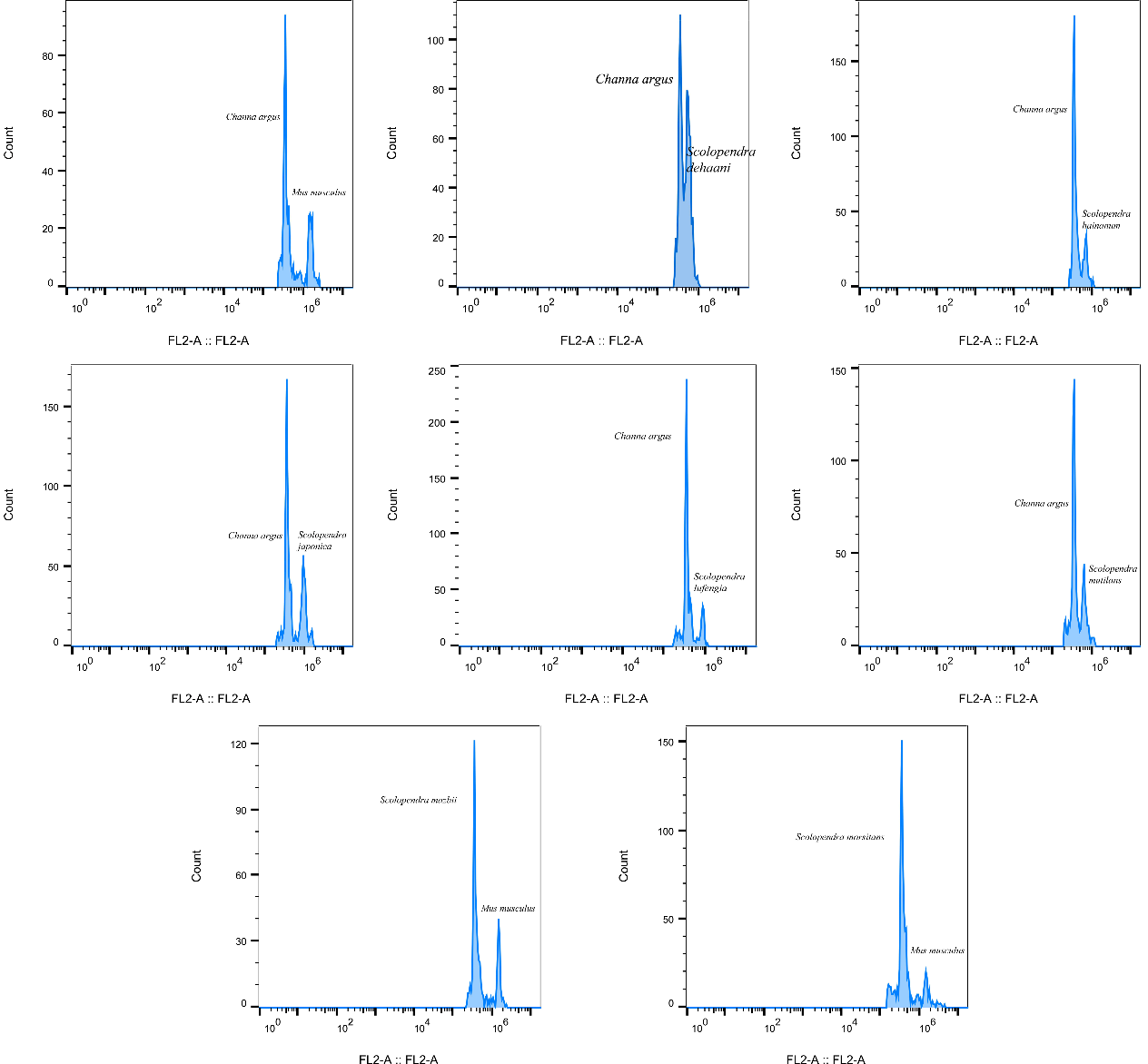
**

**Figure S3:** **Flow cytometry results plotted on mixed samples.** By mixing the reference sample with the test sample and injecting them together, a fluorescence intensity graph is generated, with the sample names labeled next to each peak.


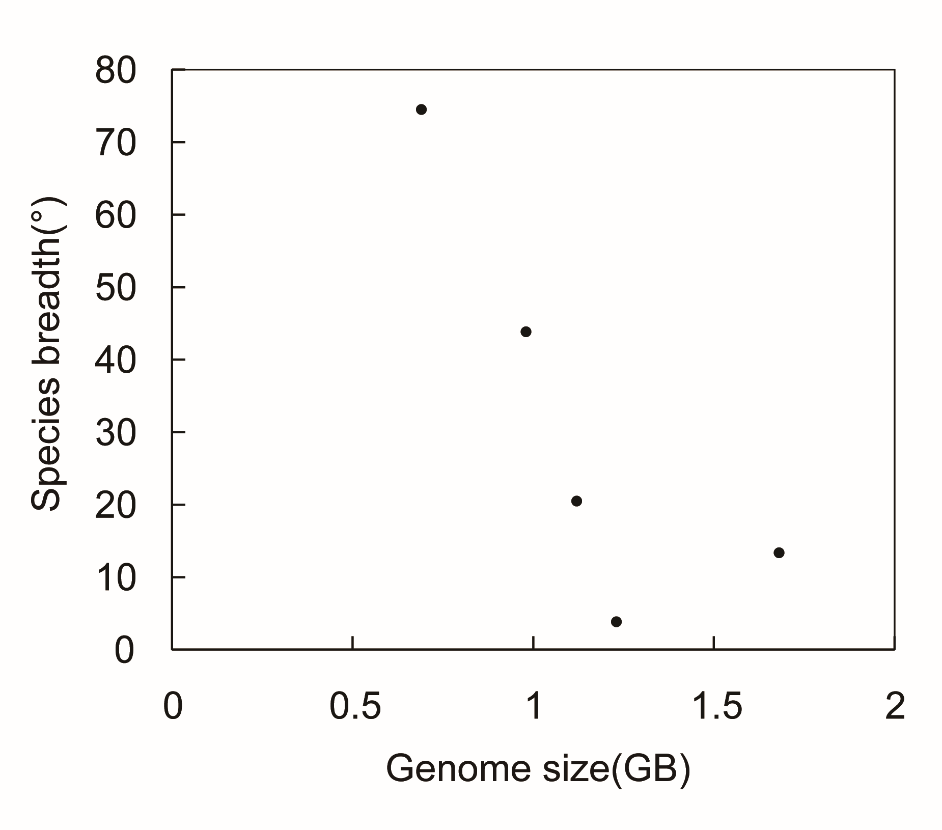


**Figure S4:** **Relationship between genome size and species distribution** **range.** The y-axis represents species distribution range (we use the max latitude minus the min latitude of all sample points for each species representative their distribution range) and the x-axis represents the genome size (GB).

**Supplementary Tables**

**Table S1**. **Sample latitude and longitude information and 19 bioclimatic variables for five filtered *Scolopendra* species.**

**Table S2.** **TNB,** **PNB and** **P-SNB for five *Scolopendra* species**

**Table S3. The relationship with phylogenetic signal between genome size and P-SNB.**

**Table S4. Statistics describing the relationships shown in Figure 2. Models were fitted using both OLS and PGLS regressions**
